# Young infant clinical signs study­­, Pakistan: a data note

**DOI:** 10.12688/gatesopenres.13317.1

**Published:** 2021-08-12

**Authors:** Shahira Shahid, Shiyam Sunder Tikmani, Nick Brown, Anita K.M. Zaidi, Fyezah Jehan, Muhammad Imran Nisar

**Affiliations:** 1 Department of Pediatrics and Child Health, Aga khan University, Karachi, Pakistan; 2 Department of Community Health Sciences, Aga Khan University, Karachi, Pakistan; 3 Uppsala University Hospital, Sweden, Sweden

**Keywords:** young infants, clinical signs, severe illness requiring hospitalization, Pakistan, community

## Abstract

Neonatal sepsis is the leading cause of child death globally with most of these deaths occurring in the first week of life. It is of utmost public health importance that clinical signs predictive of severe illness and need for referral are identified early in the course of illness. From 2002-2005, a multi country trial called the Young Infant Clinical Signs Study (YICSS) was conducted in seven sites across three South-Asian (Bangladesh, India, and Pakistan), two African (Ghana, and South Africa), and one South American (Bolivia) country. The study aimed to develop a simplified algorithm to be used by primary healthcare workers for the identification of sick young infants needing prompt referral and treatment. The main study enrolled 8,889 young infants between the ages of 0-59 days old. This dataset contains observations on 2950 young infants aged 0-59 days from the Pakistan site. The data was collected between 2003-2004 with information on the most prevalent signs and symptoms. The data from this study was used to update the Integrated Management of Childhood Illness guidelines. The World Health Organisation (WHO) seven-sign algorithm has been used in other major community-based trials to study possible serious bacterial infection and its treatment regimens.

## Introduction

In 2015, around 45% of all under-five mortality occurred in the first month of life particularly in low- and middle-income countries ^1^. Majority of neonatal deaths occur due to infections; thus, it is important to identify sick infants needing urgent referral and hospitalization. The Young Infant Clinical Signs Study (YICSS) is a multi-country study conducted across six low- and middle-income countries (Bangladesh, Bolivia, Ghana, India, South Africa, and Pakistan) at seven sites. The study determined predictive values of various clinical signs and symptoms which can be used by primary healthcare workers to identify infants with severe illness requiring hospitalization as compared to an expert pediatrician diagnosis ^2^. The study enrolled infants in two age groups: 3,177 infants in the 0–6 days age group and 5,712 infants in the 7–59 days age group. Out of the 31 signs and symptoms recorded by the community health workers, 12 identified severe illness in the first week of life and the algorithm was further reduced to seven key predictors of disease severity. The findings of this study formed the basis for updating the Integrated Management of Childhood Illness (IMCI) guidelines. Since its conception, the World Health Organisation (WHO) IMCI algorithm has been used in other major community-based studies. A multicenter observational cohort study called Aetiology of Neonatal Infection in South Asia (ANISA) and another randomised, open label, equivalence trial Simplified Antibiotic Therapy Trial (SATT) were both based on the IMCI seven-sign algorithm and proved to be important steps in understanding the infectious etiology behind neonatal possible serious bacterial infections and antibiotic regimens that can be given in case referral is not possible ^3, 4^.

The datasets presented in this paper are from the site-specific study conducted by the Aga Khan University in Karachi, Pakistan. The study aimed to validate clinical signs and symptoms in Pakistani infants in order to identify severe illness and predict hospital admissions. It was the largest site-specific cohort in the YICSS. The data is more representative of the study population since it is collected from community-based referral to primary healthcare centers. The data collection for the primary study occurred from 2003-2004. We believe these site-specific results remain relevant for similar low-and-middle-income settings. The dataset can be used by researchers to replicate the analysis or update systematic reviews and meta-analysis.

## Materials and methods

### Data description

The dataset includes 2950 observations from 1,633 infants aged 0-6 days, 817 infants aged 7-27 days and 500 infants aged 28-59 days. Infants were enrolled from September 2003 to November 2004. There are five files which are uploaded: “YICSS Dataset Version 2.xslx”, “YIS codebook Version 2.xls”, “Form A.pdf”, “Form B.pdf” and “Form C.pdf” available for download ^5^. There is a total of 253 fields in the “YIS Dataset Version 2.xlsx”. “Form A.pdf”, “Form B.pdf” and “Form C.pdf” are the study tools that were used to collect this data. A codebook “YIS codebook Version 2. xslx” gives information on individual variables.

### Data collection

The study was conducted in two peri-urban sites, Rehri Goth and Ibrahim Hyderi and an urban squatter settlement, Bilal Colony in Karachi, Pakistan. Both the peri-urban sites had Primary Healthcare Centers (PHC) run by the Aga Khan University Hospital (AKUH). All infants aged less than 60 days who were either self-referred to our center or referred by Community Health Workers (CHW) during community surveillance were first screened by a trained LHV (Lady Health Visitor) (Study Person A) after determining eligibility and taking informed consent from the parent/guardian. Form A included a questionnaire on the socio-demographic details and clinical signs which were recorded by the LHV. The infants were then referred to an experienced pediatrician (Study person B) who was blinded to the assessment of the LHV. The pediatrician determined the need for immediate referral of the infants to a tertiary care hospital, the National Institute of Child Health (NICH), based on their clinical presentation. Infant pulse and oximetry were also performed. Form B collected information of the expert paediatrician assessment and Form C described the final clinical diagnoses at the end of hospitalisation. A detailed description of the methodology has been published previously ^2, 6^.

### Data entry and management

All the forms were first checked for completion and correctness. Data was then double entered into an Epi-Data database (V.2.1, Epidata Association, Odense Denmark). Data cleaning and consistency checks were performed at the data coordination centre in Melbourne, Australia. The quality of data received from study sites was also monitored.

### Statistical methods

Following the methodology of the primary paper, we replicated the analysis for 0-6-day age group in Pakistani infants ^2^. We determined a simple association between each of the clinical signs and symptoms and the study outcome (i.e., severe illness requiring urgent referral, as confirmed by paediatrician diagnosis) using sensitivity, specificity, and odds ratio (OR) with 95% confidence intervals (CIs). We developed multiple logistic regression models to determine predictors of urgent referral. We used backward selection to identify predictors with an OR of at least 2 and 95% confidence interval excluding 1. Signs were omitted when the corresponding p-value in the multivariable model was greater than 0·05 or the adjusted OR fell below 2 and thus was considered unlikely to have major prognostic value. A further reduction of the list of signs was then made based on their prevalence and clinical judgement grounds, omitting signs that were rarely reported. All analysis was performed using Stata version 9.2 software.

### Ethics

This study was approved by the Ethics Review Committee of Aga Khan University and the Johns Hopkins University Institutional Review Board.

## Data availability

### Underlying data

Mendeley Data: YICSS Pakistan site dataset. https://doi.org/10.17632/3pgb37wck4.2 ^5^. 

This project contains the following underlying data:

1. YICSS Dataset Version 2.xlsx
2. YIS codebook Version 2.xlsx

### Extended data

Mendeley Data: YICSS Pakistan site dataset. https://doi.org/10.17632/3pgb37wck4.2 ^5^. 

This project contains the following extended data:

1. Form A.pdf (Health worker assessment)
2. Form B.pdf (Expert Pediatrician Assessment)
3. Form C.pdf (Final clinical diagnoses at the end of hospitalization)

Data are available under the terms of the Creative Commons Attribution 4.0 International license (CC-BY 4.0).

## Consent

Written informed consent for publication of the participants’ details was obtained from the parent or legal guardian of the child.
