# Supplementary material for: Young infant clinical signs study­­, Pakistan: a data note
Source: Gates Open Res. 2021 Aug 12;5:122. [Version 1] doi: 10.12688/gatesopenres.13317.1 (PMC10950895; doi:10.12688/gatesopenres.13317.1)
Supplement: Peer review history

# Reviewer report: Reviewer response for version 1

**DOI:** 10.21956/gatesopenres.14560.r35890

**Published:** 2024-07-23

**Authors:** Pascal M Lavoie

**Affiliations:** 1 Department of Pediatrics, The University of British Columbia, Vancouver, British Columbia, Canada

This study reports data from 2950 infants 0 to 59 days of age, enrolled from 3 sites in Pakistan in 2003-2004 as part of the Young Infant Clinical Signs Study. The purpose was to develop compounded clinical signs at presentation, predictive of an urgent need for referral, as defined as "infants with severe illness requiring hospitalization as compared to an expert pediatrician diagnosis". The findings of this study formed the basis for the Integrated Management of Childhood Illness (IMCI) guidelines. The data is clearly presented. I don't have major comments. However, it would be helpful to clarify:

 1) The statistical method description indicated that the analysis was replicated for the 0-6 age group in Pakistan infants. However, ref#2 refers to children under age 2 months. The data presented includes all 2950 infants, presumably with infants >6 days. I did not see a specific analysis about the 0-6 days in the current manuscript. Please clarify what this is referring to.

 2) The data is clearly labeled, but for categorical variables I was not able to access information about how the categories were defined. For example, tage refers to age groups 1, 2, etc. but the data dictionary (YIS codebook Version 2.xlsx) did not seem to specify what these categories were. It may be in the original publication, but would be useful to indicate in this specific article. For other things like "who attended delivery" I was not able to find information on how this was coded. Please clarify...

Are sufficient details of methods and materials provided to allow replication by others?

Partly

Is the rationale for creating the dataset(s) clearly described?

Yes

Are the datasets clearly presented in a useable and accessible format?

Partly

Are the protocols appropriate and is the work technically sound?

Yes

Reviewer Expertise:

Neonatology, global health research, neonatal sepsis, immunology, clinical care, epidemiology.

I confirm that I have read this submission and believe that I have an appropriate level of expertise to confirm that it is of an acceptable scientific standard, however I have significant reservations, as outlined above.

# Reviewer report: Reviewer response for version 1

**DOI:** 10.21956/gatesopenres.14560.r36061

**Published:** 2024-07-19

**Authors:** Julia Johnson

**Affiliations:** 1 Division of Neonatology, Johns Hopkins University, Baltimore, Maryland, USA

The authors previously published their analysis of predictors for severe illness in the Pakistani infants enrolled in the YICCS study (reference 6). This data note makes available the site-specific data, linked appropriately, to promote ready replication of the previously performed analyses and/or inclusion of the data in future systematic reviews and meta-analyses. The data note is written clearly, with adequate description of data collection, entry, and management, and a brief overview of methods used for the analysis.

Are sufficient details of methods and materials provided to allow replication by others?

Yes

Is the rationale for creating the dataset(s) clearly described?

Yes

Are the datasets clearly presented in a useable and accessible format?

Yes

Are the protocols appropriate and is the work technically sound?

Yes

Reviewer Expertise:

Neonatal sepsis, infection prevention and control

I confirm that I have read this submission and believe that I have an appropriate level of expertise to confirm that it is of an acceptable scientific standard.

# Reviewer report: Reviewer response for version 1

**DOI:** 10.21956/gatesopenres.14560.r35885

**Published:** 2024-03-19

**Authors:** Bijan Saha

**Affiliations:** 1 Department of Neonatology, Institute of Post Graduate Medical Education & Research and SSKM Hospital, Kolkata, West Bengal, India

This is an interesting data note from one of original center of Young infant study. 

 It has included the important signs and symptoms to diagnose underlying serious bacterial infection which will be helpful for peripheral health worker to suspect sepsis in newborn. 

 Authors have mentioned in form A  about diarrhea in the history . However, many infants are  admitted with history of  abdominal distension rather than diarrhea with sepsis and ileus.

 Form B : Secondly, they have mentioned about gestational age. Correct  assessment of gestational age is critical. Unfortunately it is not assessed correctly many a times in low and middle income countries. This needs to be highlighted. 

 Rupture of membrane : Authors have mentioned 12 hours . Traditionally it it is 18 or 24 hours. Please explain.

Are sufficient details of methods and materials provided to allow replication by others?

Yes

Is the rationale for creating the dataset(s) clearly described?

Yes

Are the datasets clearly presented in a useable and accessible format?

Yes

Are the protocols appropriate and is the work technically sound?

Yes

Reviewer Expertise:

Neonatal ventilation, Infection control and Neonatal sepsis.

I confirm that I have read this submission and believe that I have an appropriate level of expertise to confirm that it is of an acceptable scientific standard.

# Reviewer report: Reviewer response for version 1

**DOI:** 10.21956/gatesopenres.14560.r31413

**Published:** 2021-11-29

**Authors:** Salahuddin Ahmed

**Affiliations:** 1 Projahnmo Research Foundation, Dhaka, Bangladesh

The rationale is described clearly. The methods are described clearly and are replicable by others. In the introduction, the authors need to add a reference for the majority of neonatal deaths that occur due to infection. In the data collection, "Infant pulse and oximetry were also performed" is written but the most commonly used term is pulse oximetry. Which pulse oximeter was used - this needs to be added. How many days of training did LHV receive to assess children less than 60 days?

Are sufficient details of methods and materials provided to allow replication by others?

Yes

Is the rationale for creating the dataset(s) clearly described?

Yes

Are the datasets clearly presented in a useable and accessible format?

Yes

Are the protocols appropriate and is the work technically sound?

Yes

Reviewer Expertise:

Infectious disease epidemiology, technology used to diagnose disease, pneumonia

I confirm that I have read this submission and believe that I have an appropriate level of expertise to confirm that it is of an acceptable scientific standard.
